# Neuropsychiatric Symptoms and Microglial Activation in Patients with Alzheimer Disease

**DOI:** 10.1001/jamanetworkopen.2023.45175

**Published:** 2023-11-27

**Authors:** Cristiano Schaffer Aguzzoli, Pâmela C. L. Ferreira, Guilherme Povala, João Pedro Ferrari-Souza, Bruna Bellaver, Carolina Soares Katz, Hussein Zalzale, Firoza Z. Lussier, Francieli Rohden, Sarah Abbas, Douglas T. Leffa, Marina Scop Medeiros, Joseph Therriault, Andréa L. Benedet, Cécile Tissot, Stijn Servaes, Nesrine Rahmouni, Arthur Cassa Macedo, Gleb Bezgin, Min Su Kang, Jenna Stevenson, Vanessa Pallen, Ann Cohen, Oscar L. Lopez, Dana L. Tudorascu, William E. Klunk, Victor L. Villemagne, Jean Paul Soucy, Eduardo R. Zimmer, Lucas P. Schilling, Thomas K. Karikari, Nicholas J. Ashton, Henrik Zetterberg, Kaj Blennow, Serge Gauthier, Victor Valcour, Bruce L. Miller, Pedro Rosa-Neto, Tharick A. Pascoal

**Affiliations:** 1 Department of Psychiatry, School of Medicine, University of Pittsburgh, Pittsburgh, Pennsylvania; 2 Department of Neurology, University of Pittsburgh, Pittsburgh, Pennsylvania; 3 Graduate Program in Biological Sciences: Biochemistry, Universidade Federal do Rio Grande do Sul, Porto Alegre, Brazil; 4 Department of Pharmacology, Graduate Program in Biological Sciences: Pharmacology and Therapeutics, Universidade Federal do Rio Grande do Sul, Porto Alegre, Brazil; 5 Brain Institute of Rio Grande do Sul, Pontifical Catholic University of Rio Grande do Sul, Porto Alegre, RS, Brazil; 6 Department of Neurology, Pontifical Catholic University of Rio Grande do Sul, Porto Alegre, Brazil; 7 Global Brain Health Institute, University of California, San Francisco; 8 Department of Neurology, University of California, San Francisco; 9 Department of Psychiatry and Neurochemistry, Institute of Neuroscience and Physiology, the Sahlgrenska Academy at the University of Gothenburg, Mölndal, Sweden; 10 Clinical Neurochemistry Laboratory, Sahlgrenska University Hospital, Mölndal, Sweden; 11 Department of Neurodegenerative Disease, UCL Institute of Neurology, Queen Square, London, United Kingdom; 12 UK Dementia Research Institute at UCL, London, United Kingdom; 13 Hong Kong Center for Neurodegenerative Diseases, Clear Water Bay, Hong Kong, China; 14 Wisconsin Alzheimer’s Disease Research Center, University of Wisconsin School of Medicine and Public Health, University of Wisconsin–Madison; 15 Translational Neuroimaging Laboratory, McGill University Research Centre for Studies in Aging, Alzheimer’s Disease Research Unit, Douglas Research Institute, Le Centre intégré universitaire de santé et de services sociaux de l’Ouest-de-l’Île-de-Montréal; Department of Neurology and Neurosurgery, Psychiatry and Pharmacology and Therapeutics, McGill University, Montreal, Quebec, Canada; 16 Montreal Neurological Institute, McGill University, Montreal, Quebec, Canada

## Abstract

**Importance:**

Neuropsychiatric symptoms are commonly encountered and are highly debilitating in patients with Alzheimer disease. Understanding their underpinnings has implications for identifying biomarkers and treatment for these symptoms.

**Objective:**

To evaluate whether glial markers are associated with neuropsychiatric symptoms in individuals across the Alzheimer disease continuum.

**Design, Setting, and Participants:**

This cross-sectional study was conducted from January to June 2023, leveraging data from the Translational Biomarkers in Aging and Dementia cohort at McGill University, Canada. Recruitment was based on referrals of individuals from the community or from outpatient clinics. Exclusion criteria included active substance abuse, major surgery, recent head trauma, safety contraindications for positron emission tomography (PET) or magnetic resonance imaging, being currently enrolled in other studies, and having inadequately treated systemic conditions.

**Main Outcomes and Measures:**

All individuals underwent assessment for neuropsychiatric symptoms (Neuropsychiatry Inventory Questionnaire [NPI-Q]), and imaging for microglial activation ([^11^C]PBR28 PET), amyloid-β ([^18^F]AZD4694 PET), and tau tangles ([^18^F]MK6240 PET).

**Results:**

Of the 109 participants, 72 (66%) were women and 37 (34%) were men; the median age was 71.8 years (range, 38.0-86.5 years). Overall, 70 had no cognitive impairment and 39 had cognitive impairment (25 mild; 14 Alzheimer disease dementia). Amyloid-β PET positivity was present in 21 cognitively unimpaired individuals (30%) and in 31 cognitively impaired individuals (79%). The NPI-Q severity score was associated with microglial activation in the frontal, temporal, and parietal cortices (β = 7.37; 95% CI, 1.34-13.41; *P* = .01). A leave-one-out approach revealed that irritability was the NPI-Q domain most closely associated with the presence of brain microglial activation (β = 6.86; 95% CI, 1.77-11.95; *P* = .008). Furthermore, we found that microglia-associated irritability was associated with study partner burden measured by NPI-Q distress score (β = 5.72; 95% CI, 0.33-11.10; *P* = .03).

**Conclusions and Relevance:**

In this cross-sectional study of 109 individuals across the AD continuum, microglial activation was associated with and a potential biomarker of neuropsychiatric symptoms in Alzheimer disease. Moreover, our findings suggest that the combination of amyloid-β– and microglia-targeted therapies could have an impact on relieving these symptoms.

## Key Points

**Question:**

Is microglial activation, a proxy for neuroinflammation, associated with neuropsychiatric symptoms in patients with Alzheimer disease?

**Findings:**

In this cross-sectional study including 109 individuals, levels of microglial activation were associated with neuropsychiatric symptoms in individuals across the Alzheimer disease continuum. Among the neuropsychiatric symptoms, irritability was the most closely associated with the presence of activated microglia.

**Meaning:**

In this study, the abnormality of microglial activation biomarkers was associated with neuropsychiatric symptoms in patients with Alzheimer disease.

## Teaser

This cross-sectional study evaluates whether glial markers are associated with neuropsychiatric symptoms in individuals across the Alzheimer disease continuum.

## Introduction

Neuropsychiatric symptoms (NPSs) are highly prevalent in Alzheimer disease (AD) and related dementias. It is estimated that more than 80% of patients with dementia develop at least 1 NPS with severe clinical effects over the course of their illness.^1^ NPSs are associated with higher mortality and hospitalization rates, earlier institutionalization, poorer quality of life, increased caregiver distress, and higher health care expenditure in patients with Alzheimer dementia.^2,3,4^ Irritability, nighttime disturbances, and agitation are among the most common NPS manifestations in AD, with a prevalence greater than 60%.^5^ Although NPSs are clinically well characterized in living patients with Alzheimer dementia, the neurobiological underpinnings of NPSs remain elusive.

Results from recent clinical studies showing an association between amyloid-β (Aβ) and tau pathologies with NPSs have been used to support a key role of AD hallmark proteins in the development of these symptoms.^6,7,8^ Besides Aβ and tau, neuroinflammation is a prominent feature of AD, with increasing evidence suggesting that it is an early driver of disease progression.^9^ Astrocyte and microglial cells are key elements in the brain’s immune milieu, and their aberrant activation may trigger a cascade of inflammatory responses associated with the progression of cognitive decline in individuals with mild cognitive impairment (MCI) and dementia due to AD.^10,11^ Beyond its impact on AD pathogenesis, studies suggest that microglial activation is highly associated with psychosis, mania, depression, and anxiety across a spectrum of psychiatric conditions.^12^ Despite compelling evidence for a pivotal role of microglial activation in both AD physiopathology and psychiatric symptoms, it is unclear whether microglial activation is associated with NPS in the AD continuum.

In this study, we investigated the association between NPS and glial markers (microglial activation and astrocyte reactivity), in living individuals across the aging and AD continuum using the Neuropsychiatric Inventory Questionnaire (NPI-Q); positron emission tomography (PET) biomarkers of Aβ, tau, and microglial activation; and plasma glial fibrillary acidic protein (GFAP) as measures of astrocyte reactivity. Furthermore, we investigated whether the presence of glial changes is also associated with study partner and caregiver burden. By investigating the role of glial markers in NPSs, our study has potential implications for identifying predictive biomarkers and shedding light on this debilitating comorbidity. We hypothesized that glial changes would be associated with NPSs in individuals across the AD spectrum.

## Methods

### Participants

Participants were selected from the Translational Biomarkers in Aging and Dementia (TRIAD) cohort at McGill University, Canada.^13^ Recruitment was based on printed materials, word of mouth, and referrals of individuals from the community or from the McGill University Research Center for Studies in Aging outpatient clinics. Exclusion criteria included active substance abuse, major surgery, recent head trauma, safety contraindications for PET or magnetic resonance imaging (MRI), currently enrolled in other studies, and inadequately treated systemic conditions. Research participants were genotyped for the Ala147Thr variant of the *TSPO* gene (rs6971), which predicts high-, mixed-, and low-affinity binding of the [^11^C]PBR28 tracer to the 18-kDa translocator protein (TSPO).^14^ The [^11^C]PBR28 signal is negligible in individuals with low-affinity binding, while those with mixed affinity show a heterogeneous tracer signal.^9,15^ Thus, mixed- and low-affinity binders were excluded from the study to decrease the noise associated with artificial uptake variations.^16,17^ Importantly, previous studies have found no difference in AD biomarkers across different affinity groups.^18^ Individuals included in the study had PET measures for Aβ plaques ([^18^F]AZD4694), tau tangles ([^18^F]MK6240), microglial activation ([^11^C]PBR28), and a single molecule array (Simoa) plasma measure of astrocyte reactivity (plasma GFAP) at the same time point. For a detailed description of the selection of study participants, see eFigure 1 in Supplement 1. The study was approved by the Douglas Mental Health University Institute Research Ethics Board and the Montreal Neurological Institute PET Working Committee; all participants provided written informed consent. This report follows the Strengthening the Reporting of Observational Studies in Epidemiology (STROBE)^19^ reporting guideline for cross-sectional studies.

We evaluated data from 70 cognitively unimpaired (CU) and 39 cognitively impaired (CI; 25 patients with MCI, and 14 patients with Alzheimer dementia) individuals from January to June 2023. All participants underwent neuropsychological assessment, including Mini-Mental State Examination (MMSE), Neuropsychiatric Inventory Questionnaire (NPI-Q), and Clinical Dementia Rating (CDR). CU participants had a CDR of 0 and no subjective cognitive complaint. Patients with MCI had a CDR of 0.5, objective cognitive impairment, and preserved activities of daily living.^20^ Patients with Alzheimer dementia were in the mild-to-moderate dementia stage with a CDR between 0.5 and 2.0 and met the National Institute on Aging and the Alzheimer Association (NIA-AA) criteria for probable AD.^21^ All patients with Alzheimer dementia were required to have a positive Aβ-PET.

### Neuropsychiatric Assessment

All participants had an NPI-Q assessment completed by a study partner, who was either a caregiver, family member, or close friend who knew the participant well. The questionnaire provides an informant-based assessment of 12 neuropsychiatric symptoms or domains (agitation or aggression, aberrant motor behavior, irritability or lability, elation or euphoria, disinhibition, appetite or eating disturbances, apathy or indifference, delusions, hallucinations, nighttime disturbances, depression or dysphoria, and anxiety) and associated caregiver distress in research and routine clinical practice settings.^22^ The severity score is a 3-point scale of the symptom present in the patient within the last month. A score of 0 indicates no symptoms; if the symptom is present, the informant is asked to classify its severity as mild (1), moderate (2), or severe (3). The distress score is a 5-point scale associated with the impact of the patient’s symptoms on the caregiver in the past month. A score of 0 indicates no distress, while scores from 1 to 5 indicate the presence of distress with increasing severity. The NPI-Q severity score and NPI-Q distress score are the sums of individual scores from the 12 domains.

### Imaging

All 109 participants had a 3T MRI (Siemens), as well as Aβ-PET ([^18^F]AZD4694), tau-PET ([^18^F]MK6240), and microglial activation TSPO*-*PET ([^11^C]PBR28) imaging in the same brain-dedicated scanner (Siemens High-Resolution Research Tomograph). Detailed imaging methods are described in the eAppendix in Supplement 1. A global Aβ-PET standardized uptake value ratio (SUVR) was estimated from an average of SUVR in the orbitofrontal, prefrontal, anterior and posterior cingulate, temporal, precuneus, and parietal cortices.^23^ We defined Aβ-positive individuals as those with a global Aβ-PET SUVR of 1.55 or greater.^24^ Tau-PET SUVR was estimated from a temporal meta–region of interest (meta-ROI) comprising the entorhinal, hippocampus, fusiform, parahippocampal, inferior temporal, and middle temporal cortices. We considered tau-positive individuals with a temporal meta-ROI tau-PET SUVR of 1.24 or greater.^25^ Finally, for the TSPO-PET, since the topographic inflammatory response in the brain is rather unspecific, we estimated the SUVR from a composite mask of the region-wise associations between [^11^C]PBR28 and NPI-Q severity score. Microglial activation positivity was defined as the TSPO-PET SUVR values 2.5 SDs greater than the mean TSPO-PET SUVR from a separate CU young population (individuals <25 years of age), similar to what has previously been proposed.^26^ We used the whole cerebellum gray matter as the reference region for [^18^F]AZD4694 SUVRs and [^11^C]PBR28 SUVRs^27,28^ and the crus I gray matter, located in the inferior cerebellum, for [^18^F]MK6240 SUVRs.^29^ We used the Desikan-Killiany-Tourville atlas to define the ROIs.^30^

### Plasma Biomarker

Plasma GFAP concentrations were measured by Simoa using a commercial single-plex assay (No. 102336 [Quanterix]). The measurements were performed in 1 round of experiments using 1 batch of reagents by clinical scientists who were blinded to clinical data. Intra-assay coefficients of variation were less than 10%.

### Statistical Analysis

We conducted statistical models and region-wise analysis with R software version 4.2.2 (R Project for Statistical Computing). Student *t* test and contingency χ^2^ test assessed demographic differences between clinical groups for continuous and categorical variables, respectively. We used multivariable linear regression models to assess the association between TSPO-PET or GFAP and NPI-Q scores. The region-wise analysis was adjusted for age, sex, and multiple comparisons using false-discovery rate–correction at a threshold of *P* < .05. The association between TSPO-PET SUVR and NPI-Q scores was adjusted for age, sex, and cognitive status. To test the magnitude of association between biomarkers and NPSs, we transformed the PET SUVR values into *z* scores and included them as covariates. To measure the contribution of each NPI-Q severity domain to the association, we used a leave-one-out approach^31^ by iteratively removing each individual NPI-Q domain score from the total score and then compared the TSPO-PET SUVR β estimate magnitude of change. We further conducted sensitivity analyses testing the association between microglial activation and NPI-Q subscales (eFigure 3 in Supplement 1). We used the same method to assess which domains were statistically driving the association between TSPO-PET and NPS. We used the same approach to verify which NPI-Q distress domain had the greatest contribution to the model. The level of significance was set at *P* < .05 (2-tailed). Finally, we reproduced our findings using censored regression models^32^ (eTable 3 in Supplement 1) that account for the NPI-Q score floor effect.

## Results

### Participants

Our study included 109 individuals (70 CU, 25 patients with MCI, and 14 patients with Alzheimer dementia), with a median age of 71.8 years (range, 38.0-86.5 years); 72 of whom (66%) were female and 37 (34%) were male. As expected, CI individuals had significantly higher NPI-Q severity and NPI-Q distress scores, global Aβ-PET SUVR, temporal meta-ROI tau-PET SUVR, and composite TSPO-PET SUVR than CU individuals. Aβ-PET positivity was present in 21 CU individuals (30%) and in 31 CI individuals (79%). Regarding the NPI-Q severity domains, nighttime disturbances were the most prevalent (30 [28%]), followed by irritability (26 [24%]) and appetite or eating disturbances (22 [20%]). Regarding the NPI-Q distress domains, irritability was the most prevalent (18 [17%]), followed by depression (17 [16%]) and appetite or eating disturbances (14 [13%]). NPI-Q severity and distress scores for the delusion domain were 0 for all individuals. The Table presents the demographic characteristics of the study population, and eFigure 2 in Supplement 1 shows the frequency of NPI-Q severity and distress scores across the AD continuum.

**Table.** Demographic and Key Characteristics of the Study Population

Characteristic	Participants, mean (SD)		
	CU (n = 70)	MCI (n = 25)	AD dementia (n = 14)
Age, y	72 (7)	73 (5)	70 (9)
Sex, No. (%)			
Female	57 (81)	9 (36)^a^	6 (43)^a^
Male	13 (19)	16 (64)^a^	8 (57)^a^
Years of education	15 (3.7)	16 (3.1)	14 (3.6)
MMSE score	29 (0.92)	28 (1.6)	22 (6.0)^a^^,^^b^^,^^c^
CDR-SB score	0.059 (016)	1.5 (0.88)^a^^,^^c^	5.7 (2.5)^a^^,^^b^^,^^c^
APOE ε4 carriership	19 (27)	14 (56)^a^^,^^d^	7 (50)
NPI-Q severity score	0.91 (1.8)	2.4 (2.2)^a^^,^^d^	6.1 (4.7)^a^^,^^b^^,^^c^
NPI-Q distress score	0.43 (1.0)	2.3 (2.7)^a^^,^^d^	5.2 (4.8)^a^^,^^b^^,^^c^
Plasma GFAP^e^	230 (130)	230 (82)	430 (140)^a^^,^^b^^,^^c^
[^11^C]PBR28 (composite MA-PET SUVR)	1.1 (0.081)	1.1 (0.077)	1.2 (0.11)^a^^,^^b^^,^^c^
[^18^F]AZD4694 global SUVR	1.5 (0.44)	2.1 (0.62)^a^^,^^c^	2.4 (0.42)^a^^,^^c^
[^18^F]MK6240 temporal meta-ROI SUVR	0.88 (0.12)	1.2 (0.51)^a^^,^^d^	2.1 (0.98)^a^^,^^b^^,^^c^
Biomarker status, No. (%)			
MA positive	34 (49)	15 (60)	12 (86)^a^^,^^d^
Aβ positive	21 (30)	17 (68)^a^^,^^d^	14 (100)^a^^,^^b^^,^^c^
Tau positive	2 (3)	7 (28)^a^^,^^d^	10 (71)^a^^,^^b^^,^^c^

Abbreviations: Aβ, amyloid-β; AD, Alzheimer disease; CDR-SB, Clinical Dementia Rating–Sum of Boxes; CU, cognitively unimpaired; GFAP, glial fibrillary acidic protein; MA, microglial activation; MCI, mild cognitive impairment; MMSE, Mini Mental State Examination; NPI-Q, Neuropsychiatry Inventory Questionnaire; PET, positron emission tomography; ROI, region of interest; SUVR, standardized uptake value ratio.

^a^ Tukey correction for multiple comparisons tested significant differences from CU.

^b^ Tukey correction for multiple comparisons tested significant differences from MCI.

^c^ *P* < .001. *P* values indicate the analysis of variance results on the differences between groups. A contingency χ^2^ test was performed for sex, APOE e4 status, and CDR.

^d^ *P* < .05. *P* values indicate the analysis of variance results on the differences between groups. A contingency χ^2^ test was performed for sex, APOE e4 status, and CDR.

^e^ Data were available for 52 participants in the CU group; 19, MCI group; and 12, AD group.

### Regional Microglial Activation and NPS

**Figure 1.**
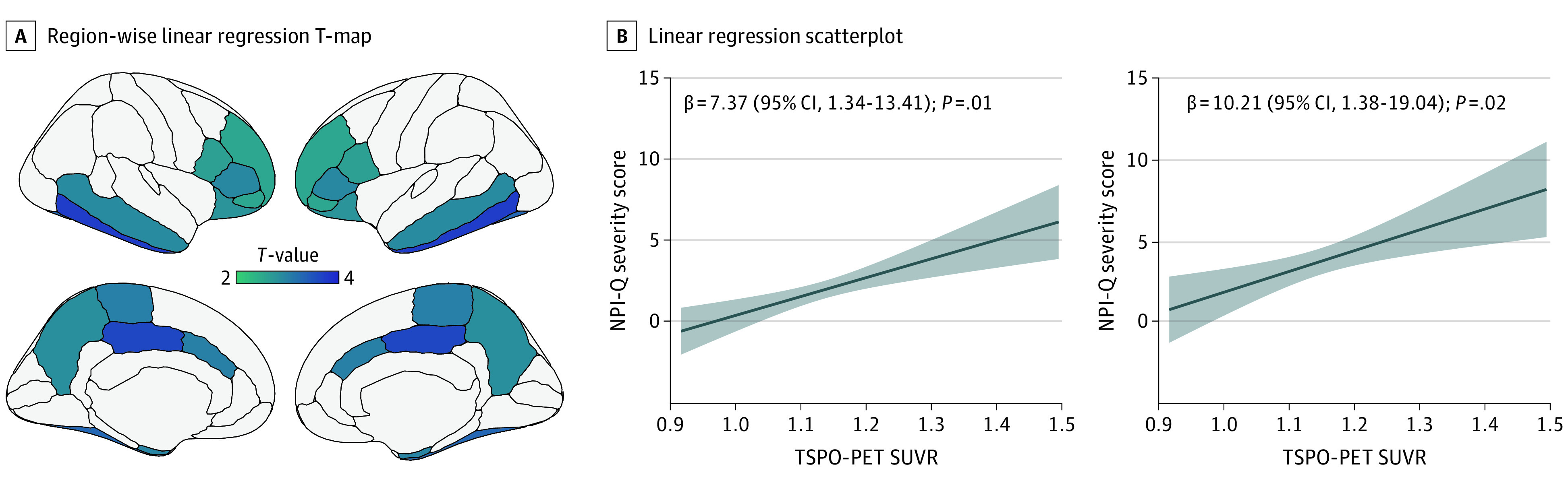
Microglial Activation and Neuropsychiatric Symptoms Across the Alzheimer Disease Continuum A, Region-wise linear regression T-map shows an association of [^11^C]PBR28 standardized uptake value ratio (SUVR) and Neuropsychiatry Inventory Questionnaire (NPI-Q) severity score in the inferior temporal, posterior cingulate, fusiform, paracentral, caudal anterior cingulate, entorhinal, middle temporal, pars triangularis, precuneus, lateral orbitofrontal, pars opercularis, pars orbitalis, and rostral middle frontal cortex. B, Linear regression scatterplot shows the association of 18-kDa translocator protein (TSPO) positron emission tomography (PET) SUVR and NPI-Q severity score in the entire population, and in individuals presenting with NPI-Q severity score of at least 1.

**Figure 2.**
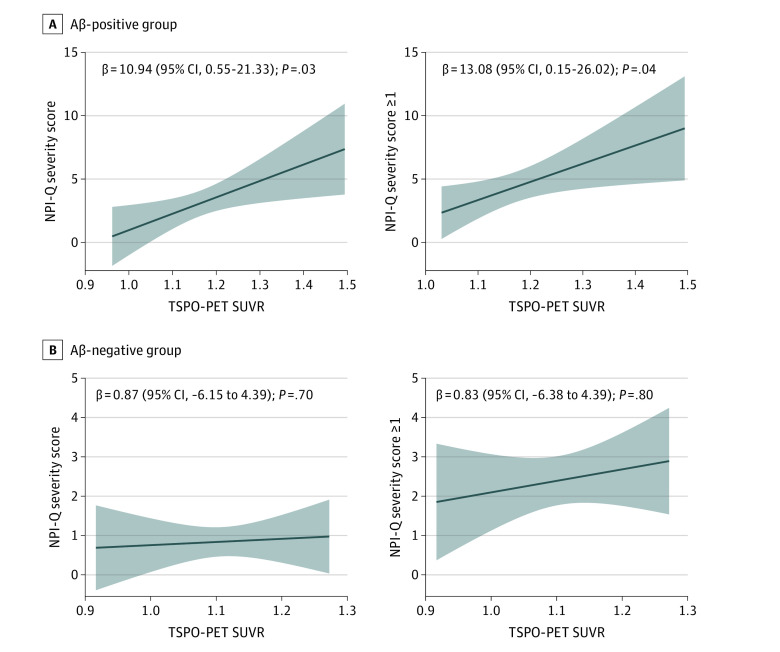
Microglial Activation, Neuropsychiatric Symptoms, and Amyloid-β (Aβ) Linear regression scatterplots illustrate the association between microglial activation and neuropsychiatric symptoms in the group of individuals presenting as Aβ positive and Aβ negative. NPI-Q indicates Neuropsychiatry Inventory Questionnaire; PET, positron emission tomography; SUVR, standardized uptake value ratio; TSPO, 18-kDa translocator protein.

Region-wise linear regression analysis revealed significant positive associations between microglial activation and NPI-Q severity score in the inferior temporal, posterior cingulate, fusiform, paracentral, caudal anterior cingulate, entorhinal, middle temporal, pars triangularis, precuneus, lateral orbitofrontal, pars opercularis, pars orbitalis, and rostral middle frontal cortices (Figure 1A; eTable 1 in Supplement 1). These associations survived correction for Aβ, tau, and cognitive status across the entire population (β = 7.37; 95% CI, 1.34-13.41; *P* = .01; *R*^2^ = 0.26) (eTable 2 in Supplement 1) and when excluding individuals presenting NPI-Q severity scores of 0 (β = 10.21; 95% CI, 1.38-19.04; *P* = .02; *R*^2^ = 0.15) (Figure 1B). These associations remained significant when restricting the analysis to the Aβ-positive group across the entire population (β = 10.94; 95% CI, 0.55-21.33; *P* = .03; *R*^2^ = 0.17) and in the individuals presenting Aβ-positive and with NPI-Q severity scores of at least 1 (β = 13.08; 95% CI, 0.15-26.02; *P* = .04; *R*^2^ = 0.10) but not when restricting the analysis in the Aβ-negative group (Figure 2A-B). These associations were not significant when applying an interaction of TSPO-PET with cognitive status (model: NPI severity score as a function of TSPO-PET × cognitive status + age + sex; β = 5.10; 95% CI, −6.93 to 17.14; *P* = .40, *R*^2^ = 0.24), which suggests that these results were not driven by any clinical group. Alternatively, these results may also be due to small differences in TSPO SUVR between groups. Interestingly, we did not find significant associations between NPI-Q severity score and astrocyte reactivity measured by plasma GFAP (83 participants; β = 0.004; 95% CI, −0.0007 to 0.009; *P* = .09, *R*^2^ = 0.21).

### Microglial Activation and Specific NPSs

**Figure 3.**
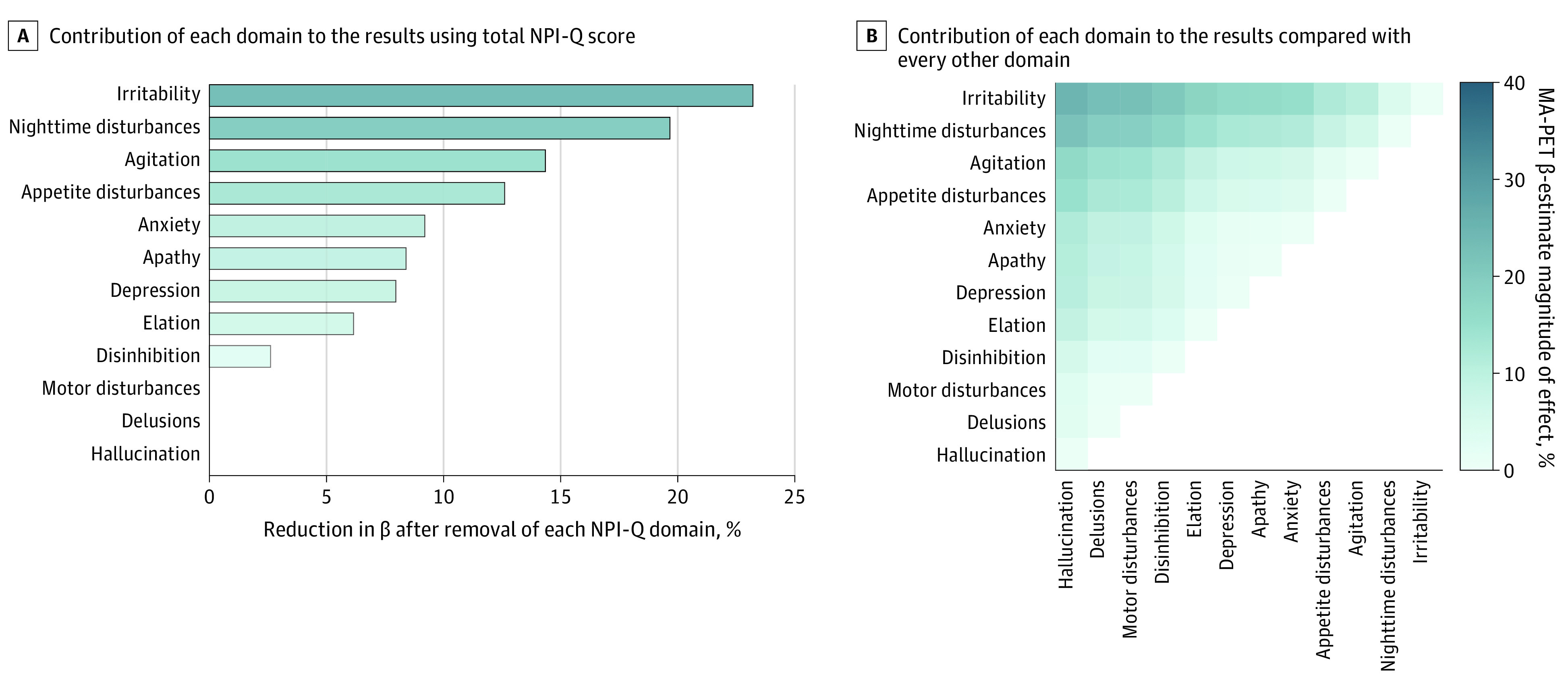
Contribution of Neuropsychiatry Inventory Questionnaire (NPI-Q) Domains to Microglial Activation (MA) Across the Alzheimer Disease Continuum A, Bars show NPI-Q domains’ contributions to the association between microglial activation and NPI-Q severity score. B, Heat map shows contributions of NPI-Q domains compared with every other domain. PET indicates positron emission tomography.

Leave-one-out analysis revealed that irritability had the greatest contribution (22.8%), followed by nighttime disturbance (19.3%), agitation (14.1%), and appetite or eating disturbances (12.4%) to the association between NPSs and microglial activation (Figure 3A-B; eTable 4 in Supplement 1).

### Microglial Activation, Irritability, and Study Partner Distress

**Figure 4.**
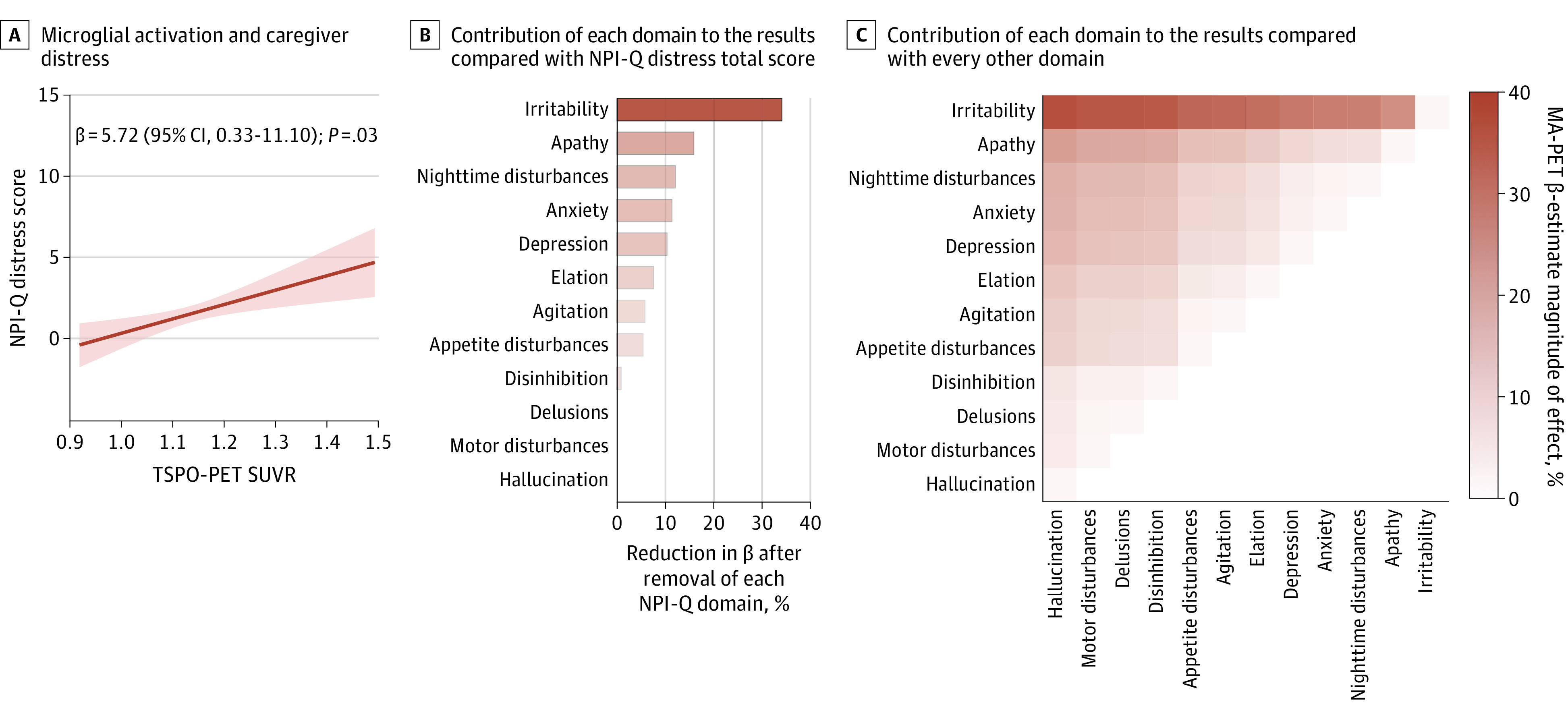
Microglial Activation-Associated Irritability and Study Partner or Caregiver Distress A, Linear regression scatterplot shows the association between microglial activation and study partner or caregiver distress. B, Bars show contributions of Neuropsychiatry Inventory Questionnaire (NPI-Q) domains to study partner or caregiver burden. C, Heat map shows NPI-Q contributions to model compared with every other domain. MA indicates microglial activation; PET, positron emission tomography; SUVR, standardized uptake value ratio; TSPO, 18-kDa translocator protein.

Microglial activation showed a significant association with NPI-Q distress score (β = 5.72; 95% CI, 0.33-11.10; *P* = .03; *R*^2^ = 0.26) (Figure 4A). Using the leave-one-out technique, we found that microglial-associated irritability had the greatest contribution (33.95%) to the study partner or caregiver distress compared with other NPI-Q distress domains (Figure 4B-C) (eTable 5 in Supplement 1).

## Discussion

We found that microglial activation is associated with NPSs in individuals across the AD spectrum. Furthermore, we found that irritability, nighttime disturbances, and agitation are the NPS domains most likely to be associated with microglial activation in the human brain.

We found that microglial activation is associated with NPS in individuals across the AD continuum. Interestingly, NPS did not significantly correlate with astrocyte reactivity measured by GFAP, suggesting possible specificity to a microglia immune cell type. These results support previous studies suggesting that microglial activation plays an important role in the development of psychiatric symptoms such as mania, depression, psychosis, and anxiety in a range of psychiatric disorders.^33,34^ Recent studies have linked Aβ and tau with NPSs across the AD spectrum,^6,7,35^ and we also found a direct association between Aβ or tau with NPS. However, when we added microglial activation to the models, this measure showed an independent contribution to NPS dysfunction. These findings suggest microglial activation as a potential biomarker, complementary to Aβ or tau, capable of identifying individuals across the AD spectrum who are most likely to exhibit NPSs.

Our results suggest that microglial activation predominantly exacerbates irritability, nighttime disturbances, and agitation. Conversely, microglial activation exerts less influence on the development of motor disturbances and hallucinations. These results are part of growing evidence suggesting a detrimental effect of microglial activation in the early stages of disease progression (ie, the transition from MCI to early AD dementia),^9^ which has also been shown to be the stage when irritability, nighttime disturbances, and agitation tend to appear.^36^ Conversely, motor disturbances and hallucinations appear in more severe dementia stages and may be driven by other pathologies. Our data also suggest that mood symptoms (depression and anxiety) could potentially be associated with factors other than microglial activation in the AD clinical spectrum. However, animal studies support a deleterious effect of microglial activation on NPS, such as anxiety and depression symptoms.^37,38^ Altogether, these results support the notion that AD clinical trials testing drugs targeting microglial activation could use NPS, such as irritability and agitation, as secondary outcomes.

The association between microglial activation and NPS was identified predominantly in the posterior cingulate, precuneus, inferior temporal, and anterior cingulate cortices. The posterior cingulate and precuneus, and to a lesser extent, the middle lateral temporal cortices, are described as seed regions of the default mode network (DMN), which is implicated in self-referential mental activity. This network is disrupted in patients with AD.^39,40,41^ Dysfunction in the DMN is associated with early accumulation of Aβ in the brain and with the appearance of agitation, depression, and anxiety.^42,43^ In addition, microglial activation in the inferior temporal, fusiform, and entorhinal cortices (key regions for tau accumulation in early Braak stages) is associated with NPS. Recent studies show that tau deposition in the temporal lobe is associated with depression, apathy, and nighttime disturbances.^8,44^ Finally, we found that microglial activation was associated with NPS in the anterior cingulate cortex, which is postulated as a central node of the salience network and has been associated with agitation and irritability in AD.^45,46^ Notably, the topographic localization of our results overlapped with brain circuits typically impaired in AD and associated with NPS development.

Also, the presence of brain microglial activation in the participants was associated with the study partner and caregiver distress. It is established that NPSs have a profound negative effect on caregivers’ quality of life, thus contributing to the overall burden.^47^ We found that microglia-associated irritability was associated with the study partners or caregivers’ distress, alongside cognitive impairment. The fact that previous studies have shown that irritability is a major cause of caregivers’ distress,^48^ early placement in long-term health facilities, morbidity, and mortality in patients support a clinical relevance for our findings.^49^ Another interesting finding was that microglia-associated apathy exerted a lower influence on patients’ NPS severity than on partner or caregiver distress. This dissociation is in line with studies showing that apathy leads to disproportionally elevated levels of distress in caregivers, mediated by the challenge of disengagement coping strategies.^50,51^ Altogether, these results suggest that the impact of microglial activation across the AD continuum extends beyond the development of NPSs and indirectly heightens study partner or caregiver burden.

### Limitations

Our study has some limitations. The NPI-Q is a shorter and simpler version of NPI that may not capture NPS frequency and subtle changes in the patient’s behavior. The low prevalence of motor disturbances and hallucinations and absence of delusions in our cohort reflect relatively mild to moderate disease stages. Studies using larger population-based cohorts across the AD spectrum, encompassing the full range of AD stages from preclinical to severe dementia, are desirable to increase the generalizability of our findings. Furthermore, research designed to identify specific inflammation-related proteins linked to our findings could potentially lead to cerebral spinal fluid and possibly blood biomarkers that could have a greater clinical utility to track NPSs in the future.

## Conclusions

In conclusion, our results support that microglial activation plays a key role in the development of NPSs for individuals on the AD continuum. These results suggest that microglial activation biomarkers can be useful in identifying NPSs and that developing drugs targeting microglial activation could potentially alleviate NPSs in patients with AD.

## Notes

Supplement 1. **eAppendix.** Detailed Imaging Methods **eFigure 1.** Study Participant Flowchart **eFigure 2.** Frequency of NPI-Q Severity and Distress Scores **eFigure 3.** Microglial Activation and the Agitation and Frontal NPI-Q Subscales **eTable 1.** TSPO-PET β-Estimate and Associated Confidence Interval, T-Values, and Adjusted P-Values From the Significant Region-Wise Associations **eTable 2.** Microglial Activation and Neuropsychiatric Dysfunction **eTable 3.** Microglial Activation and Neuropsychiatric Dysfunction Using a Censored Regression Model **eTable 4.** TSPO-PET β-Estimate, Confidence Interval, T-Value, P-Value, and the Magnitude of Change of Each NPI-Q Severity Domain When Applying the Leave-One-Out Technique **eTable 5.** TSPO-PET β-Estimate, Confidence Interval, T-Value, P Value, and the Magnitude of Change of Each NPI-Q Distress Domain When Applying the Leave-One-Out Technique
eReferences.

Supplement 2.
Data Sharing Statement
