# Supplementary material for: Neuropsychiatric Symptoms and Microglial Activation in Patients with Alzheimer Disease
Source: JAMA Netw Open. 2023 Nov 27;6(11):e2345175. doi: 10.1001/jamanetworkopen.2023.45175 (PMC10682836; doi:10.1001/jamanetworkopen.2023.45175)
Supplement: Supplement 1. — eAppendix. Detailed Imaging Methods eFigure 1. Study Participant Flowchart eFigure 2. Frequency of NPI-Q Severity and Distress Scores eFigure 3. Microglial Activation and the Agitation and Frontal NPI-Q Subscales eTable 1. TSPO-PET β-Estimate and Associated Confidence Interval, T-Values, and Adjusted P-Values From the Significant Region-Wise Associations eTable 2. Microglial Activation and Neuropsychiatric Dysfunction eTable 3. Microglial Activation and Neuropsychiatric Dysfunction Using a Censored Regression Model eTable 4. TSPO-PET β-Estimate, Confidence Interval, T-Value, P-Value, and the Magnitude of Change of Each NPI-Q Severity Domain When Applying the Leave-One-Out Technique eTable 5. TSPO-PET β-Estimate, Confidence Interval, T-Value, P Value, and the Magnitude of Change of Each NPI-Q Distress Domain When Applying the Leave-One-Out Technique eReferences. [file jamanetwopen-e2345175-s001.pdf]

Supplemental Online Content

Aguzzoli CS, Ferreira PL, Povala G, et al. Neuropsychiatric symptoms and microglial activation in patients with Alzheimer disease. *JAMA Netw Open.* 2023;6(11):e2345175. doi:10.1001/jamanetworkopen.2023.45175

eAppendix. Detailed Imaging Methods

eFigure 1. Study Participant Flowchart

eFigure 2. Frequency of NPI-Q Severity and Distress Scores

eFigure 3. Microglial Activation and the Agitation and Frontal NPI-Q Subscales

eTable 1. TSPO-PET β-Estimate and Associated Confidence Interval, T-Values, and Adjusted P-Values From the Significant Region-Wise Associations

eTable 2. Microglial Activation and Neuropsychiatric Dysfunction

eTable 3. Microglial Activation and Neuropsychiatric Dysfunction Using a Censored Regression Model

eTable 4. TSPO-PET β-Estimate, Confidence Interval, T-Value, P-Value, and the Magnitude of Change of Each NPI-Q Severity Domain When Applying the Leave-One-Out Technique

eTable 5. TSPO-PET β-Estimate, Confidence Interval, T-Value, P Value, and the Magnitude of Change of Each NPI-Q Distress Domain When Applying the Leave-One-Out Technique

eReferences.

This supplemental material has been provided by the authors to give readers additional information about their work.

© 2023 Aguzzoli CS et al. *JAMA Network Open.*

eAppendix. Detailed Imaging Methods

Aβ-PET, tau-PET, and microglial activation PET were acquired 40-70 min, 90-110 min, and 60-90 min post injection, respectively. Radiosynthesis of PET tracers have been described elsewhere [1-3]. The mean (SD) injected dose was 240.3 (20.9) megabecquerel (MBq) for [^{18}F]AZD4694; 228.8 (34.7) MBq for [^{18}F]MK6240; and 384 (17) MBq for [^{11}C]PBR28. Aβ-PET, tau-PET, and MA-PET scans were reconstructed using the ordered subset expectation maximization (OSEM) algorithm on a 4D volume with three frames (3 × 600 s), four frames (4 × 300 s), and six frames (6 x 300 s) respectively. PET images were corrected for motion, dead time, decay, and scattered and random coincidences, and were registered to the native T1-weighted MRI, which was registered to the ADNI space. PET images were spatially smoothed to an 8-mm full-width at half maximum resolution.

© 2023 Aguzzoli CS et al. *JAMA Network Open.*

eFigure 1. Study Participant Flowchart

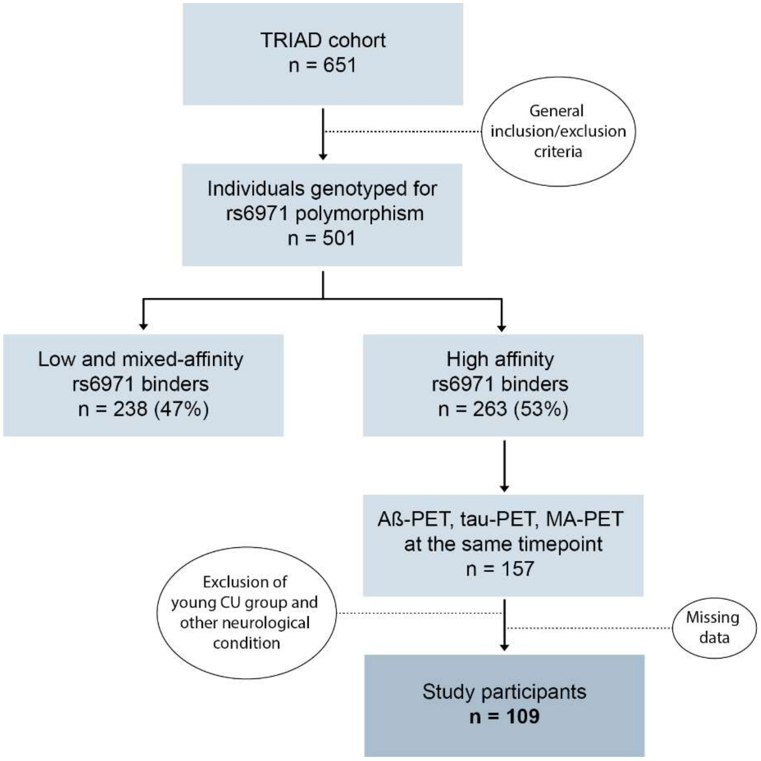

© 2023 Aguzzoli CS et al. *JAMA Network Open.*

eFigure 2. Frequency of NPI-Q Severity and Distress Scores

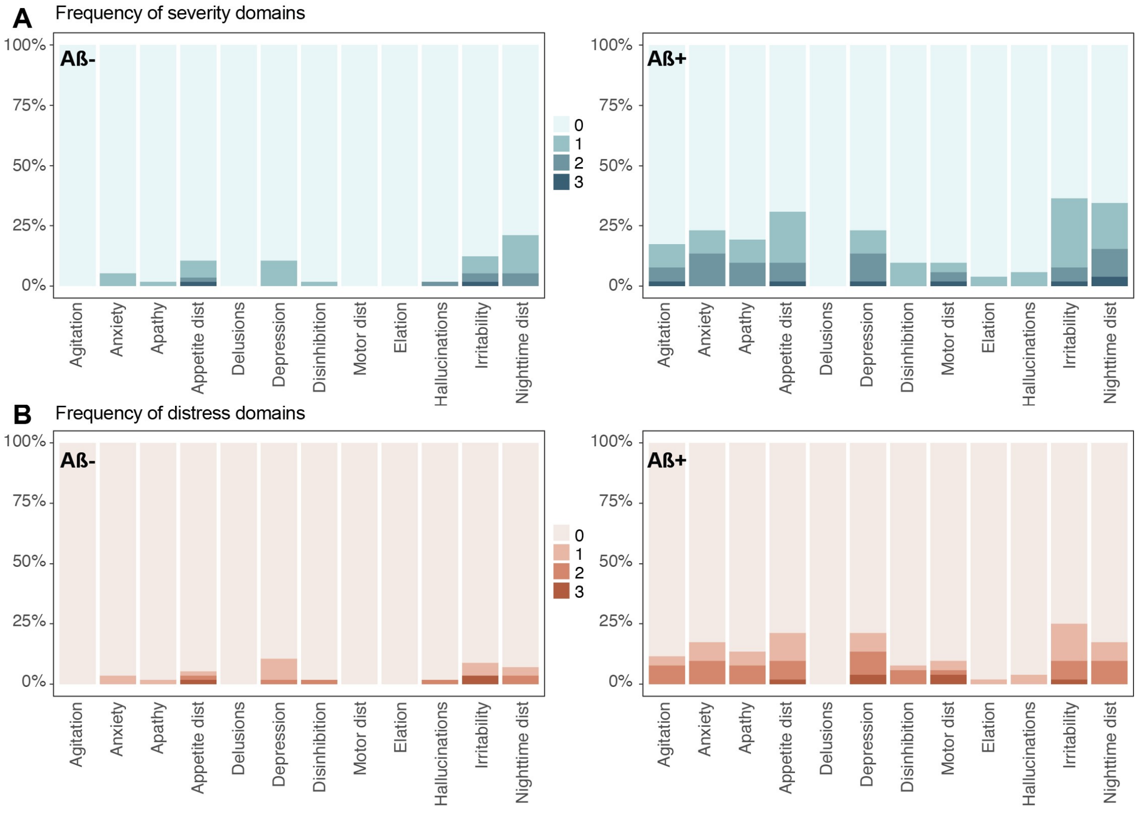

(A) Bars show the frequency of NPI-Q severity scores in Aβ negative and positive groups. (B) Bars show the frequency of NPI-Q distress scores in Aβ negative and positive groups

© 2023 Aguzzoli CS et al. *JAMA Network Open.*

eFigure 3. Microglial Activation and the Agitation and Frontal NPI-Q Subscales

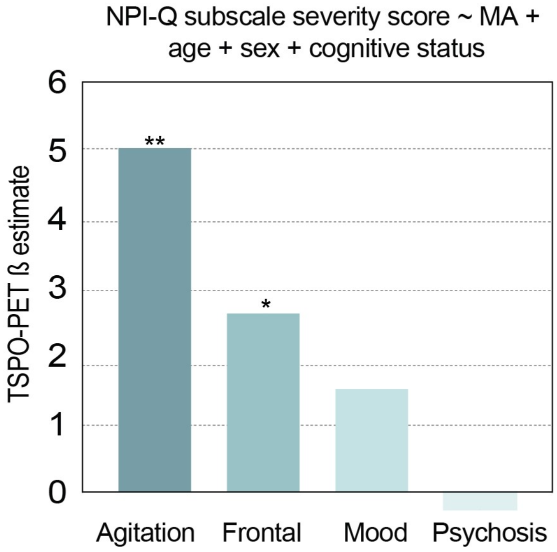

We grouped the NPI-Q in 4 subscales as described in previous studies[4]: agitation (agitation, aberrant motor behavior, irritability, nighttime disturbances), frontal (appetite abnormalities, apathy, disinhibition, elation), mood (depression, anxiety), and psychosis (delusion, hallucinations). To examine the association between microglial activation and NPI-Q subscales, we assessed the sum of each of these 4 subscales as a function of regional TSPO-PET SUVR adjusting for age, sex, and cognitive status. Our results show that microglial activation is associated with the agitation ($\beta = 5.03$, C.I = 1.73 - 8.33, $P = 0.003$, $R^2 = 0.14$) and the frontal ($\beta = 2.60$, C.I = 0.37 - 4.84, $P = 0.02$, $R^2 = 0.22$), but not with the psychosis or the mood subscales.

© 2023 Aguzzoli CS et al. *JAMA Network Open.*

eTable 1. TSPO-PET β-Estimate and Associated Confidence Interval, *T*-Values, and Adjusted *P*-Values From the Significant Region-Wise Associations

Brain region	TSPO-PET β-estimate	Confidence interval	T-value	P-value	Adjusted P-value*
Inferior temporal	12.350	5.82 - 18.88	3.75	<.001	0.011
Posterior cingulate	7.411	3.38 - 11.44	3.646	<.001	0.007
Fusiform gyrus	11.622	4.61 - 18.63	3.288	0.001	0.017
Paracentral cortex	7.089	2.45 - 11.72	3.033	0.003	0.028
Caudal anterior cingulate	6.002	2.06 - 9.94	3.02	0.003	0.024
Entorhinal cortex	7.596	2.53 - 12.66	2.973	0.003	0.023
Pars triangularis	6.050	1.92 - 10.17	2.910	0.004	0.023
Middle temporal	8.952	2.79 - 15.10	2.883	0.004	0.022
Precuneus	8.173	2.46 - 13.87	2.84	0.005	0.022
Lateral orbitofrontal	7.993	2.25 - 13.72	2.763	0.006	0.025
Pars opercularis	6.471	1.57 - 11.36	2.622	0.010	0.034
Pars orbitalis	6.362	1.41 - 11.30	2.552	0.012	0.038
Rostral middle frontal	5.805	1.27 - 10.33	2.539	0.012	0.036

* Adjusted for multiple comparisons with false discovery rate

© 2023 Aguzzoli CS et al. *JAMA Network Open.*

eTable 2. Microglial Activation and Neuropsychiatric Dysfunction

Variable	β (95% confidence interval)	*T*-value	*P*-value
Model: NPI-Q severity score ~ [^{11}C]PBR28 SUVR + [^{18}F]AZD4694 SUVR + [^{18}F]MK6240 SUVR + age + sex + cognitive status			
[^{11}C]PBR28 SUVR	0.68 (0.12 - 1.29)	2.42	0.01
[^{18}F]AZD4694 SUVR	0.29 (-0.36 - 0.94)	0.88	0.37
[^{18}F]MK6240 SUVR	0.53 (-0.23 - 1.30)	1.38	0.16
Age	-0.40 (-1.80 - 0.99)	-0.577	0.565
Sex (female)	0.34 (-1.23 – 2.73)	0.573	0.567
**Cognitive status**			
CI	1.71 (0.25 – 3.16)	2.33	0.02

© 2023 Aguzzoli CS et al. *JAMA Network Open.*

eTable 3. Microglial Activation and Neuropsychiatric Dysfunction Using a Censored Regression Model

**Variable**	**β (95% confidence interval)**	***T*-value**	***P*-value**
Censored regression Tobit model: NPI-Q severity score ~ [^{11}C]PBR28 SUVR + [^{18}F]AZD4694 SUVR + [^{18}F]MK6240 SUVR + age + sex + cognitive status			
[^{11}C]PBR28 SUVR	1.10 (0.13 – 2.06)	2.24	0.02
[^{18}F]AZD4694 SUVR	0.43 (-0.68 - 1.54)	0.75	0.45
[^{18}F]MK6240 SUVR	0.55 (-0.60 - 1.71)	0.93	0.35
Age	0.15 (-2.34 - 2.66)	0.12	0.90
Sex (female)	0.80 (-1.19 – 2.79)	0.79	0.42
**Cognitive status**			
CI	3.48 (1.11 – 5.85)	2.88	0.003

© 2023 Aguzzoli CS et al. *JAMA Network Open.*

eTable 4. TSPO-PET β-Estimate, Confidence Interval, *T*-Value, *P*-Value, and the Magnitude of Change of Each NPI-Q Severity Domain When Applying the Leave-One-Out Technique

Removed domain	TSPO-PET β-estimate	Confidence interval	*T*-value	*P*-value	TSPO-PET β-estimate magnitude of change (%)
Irritability	6.866	1.77 - 11.95	2.673	0.008	22.847
Nighttime	7.175	2.11 - 12.24	2.810	0.005	19.372
Agitation	7.640	2.17 - 13.10	2.774	0.006	14.143
Appetite	7.795	2.55 - 13.03	2.948	0.003	12.411
Anxiety	8.092	2.77 - 13.41	3.018	0.003	9.072
Apathy	8.162	2.79 - 13.52	3.016	0.003	8.285
Depression	8.200	3.20 - 13.19	3.256	0.001	7.859
Elation	8.360	2.56 - 14.15	2.861	0.005	6.055
Disinhibition	8.670	2.92 - 14.41	2.991	0.003	2.573
Motor disturbance	8.881	3.19 - 14.56	3.097	0.002	0.203
None	8.899	3.01 - 14.78	2.999	0.003	0
Delusions	8.899	3.01 - 14.78	2.999	0.003	0
Hallucination	9.151	3.28 - 15.02	3.091	0.002	-2.825

Model: [NPI-Q severity score (removing one domain)] ~ TSPO-PET + sex + age + cognitive status

© 2023 Aguzzoli CS et al. *JAMA Network Open.*

eTable 5. TSPO-PET β-Estimate, Confidence Interval, *T*-Value, *P* Value, and the Magnitude of Change of Each NPI-Q Distress Domain When Applying the Leave-One-Out Technique

Removed domain	TSPO-PET β-estimate	Confidence interval	*T*-value	*P*-value	TSPO-PET β-estimate magnitude of change (%)
Irritability	3.778	-1.00 - 8.55	1.568	0.120	33.958
Apathy	4.816	-0.12 - 9.76	1.932	0.056	15.819
Nighttime	5.035	0.06 - 10.00	2.008	0.047	11.983
Anxiety	5.074	0.30 - 9.84	2.111	0.037	11.314
Depression	5.132	0.59 - 9.67	2.243	0.027	10.287
Elation	5.289	-0.04 - 10.62	1.966	0.051	7.543
Agitation	5.393	0.44 - 10.33	2.163	0.032	5.732
Appetite	5.415	0.68 - 10.14	2.269	0.025	5.347
Disinhibition	5.672	0.56 - 10.78	2.201	0.029	0.847
None	5.721	0.33 - 11.10	2.108	0.037	0
Delusions	5.721	0.33 - 11.10	2.108	0.037	0
Motor disturbance	5.737	0.61 - 10.86	2.219	0.028	-0.281
Hallucination	5.867	0.46 - 11.27	2.152	0.033	-2.550

Model: [NPI-Q distress score (removing one domain)] ~ TSPO-PET + sex + age + cognitive status

© 2023 Aguzzoli CS et al. *JAMA Network Open.*

eReferences

1. Cselényi Z, Jönhagen ME, Forsberg A, et al. Clinical validation of 18F-AZD4694, an amyloid-β-specific PET radioligand. *J Nucl Med*. Mar 2012;53(3):415-24. doi:10.2967/jnumed.111.094029
2. Pascoal TA, Shin M, Kang MS, et al. In vivo quantification of neurofibrillary tangles with [(18)F]MK-6240. *Alzheimers Res Ther*. Jul 31 2018;10(1):74. doi:10.1186/s13195-018-0402-y
3. Zanotti-Fregonara P, Pascual B, Rizzo G, et al. Head-to-Head Comparison of (11)C-PBR28 and (18)F-GE180 for Quantification of the Translocator Protein in the Human Brain. *J Nucl Med*. Aug 2018;59(8):1260-1266. doi:10.2967/jnumed.117.203109
4. Trzepacz PT, Saykin A, Yu P, et al. Subscale validation of the neuropsychiatric inventory questionnaire: comparison of Alzheimer's disease neuroimaging initiative and national Alzheimer's coordinating center cohorts. *Am J Geriatr Psychiatry*. Jul 2013;21(7):607-22. doi:10.1016/j.jagp.2012.10.027

© 2023 Aguzzoli CS et al. *JAMA Network Open.*
