# Supplementary material for: Neuropsychiatric Symptoms and Microglial Activation in Patients with Alzheimer Disease
Source: JAMA Netw Open. 2023 Nov 27;6(11):e2345175. doi: 10.1001/jamanetworkopen.2023.45175 (PMC10682836; doi:10.1001/jamanetworkopen.2023.45175)
Supplement: Supplement 2. — Data Sharing Statement [file jamanetwopen-e2345175-s002.pdf]

Data Sharing Statement

Schaffer Aguzzoli. Neuropsychiatric Symptoms and Microglial Activation in Patients with Alzheimer Disease. *JAMA Netw Open*. Published November 27, 2023. doi:10.1001/jamanetworkopen.2023.45175

Data
Data available: Yes
Data types: Deidentified participant data
How to access data: cristiano.aguzzoli@gbhi.org pascoalt@upmc.edu
When available: With publication

Supporting Documents
Document types: None

Additional Information
Who can access the data: Researchers whose proposed use of the data has been approved
Types of analyses: For an academic purpose
Mechanisms of data availability: With a signed data access agreement
